# Supplementary material for: eIF4E3 forms an active eIF4F complex during stresses (eIF4FS) targeting mTOR and re-programs the translatome
Source: Nucleic Acids Res. 2021 Apr 24;49(9):5159–76. doi: 10.1093/nar/gkab267 (PMC8136781; doi:10.1093/nar/gkab267)
Supplement: gkab267_Supplemental_File [file gkab267_supplemental_file.pdf]

Supporting material

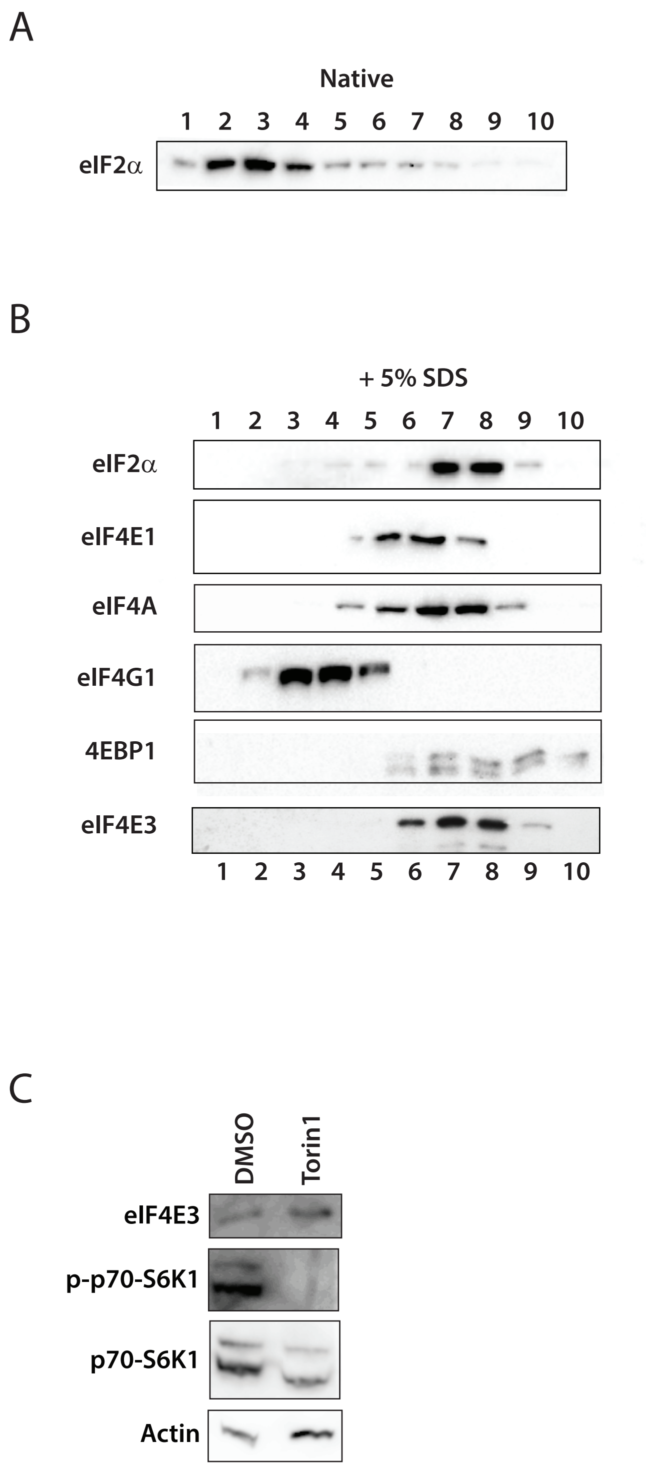

Figure S1. Glycerol gradients can be used to follow the eIF4F complex assembly and the expression of eIF4E3 does not change during Torin1 treatment. A. Western blots showing the sedimentation profiles on glycerol gradients of eIF2α from HEK293T cell lysates. **B.** Western blots showing the sedimentation profiles on glycerol gradients of members of the eIF4F complex, 4EBP1 and eIF2α from HEK293T cell lysates treated with 5% SDS and heated at 65°C. **C.** Western blots showing eIF4E3 and members in HEK293T cells after treatment with Torin1.

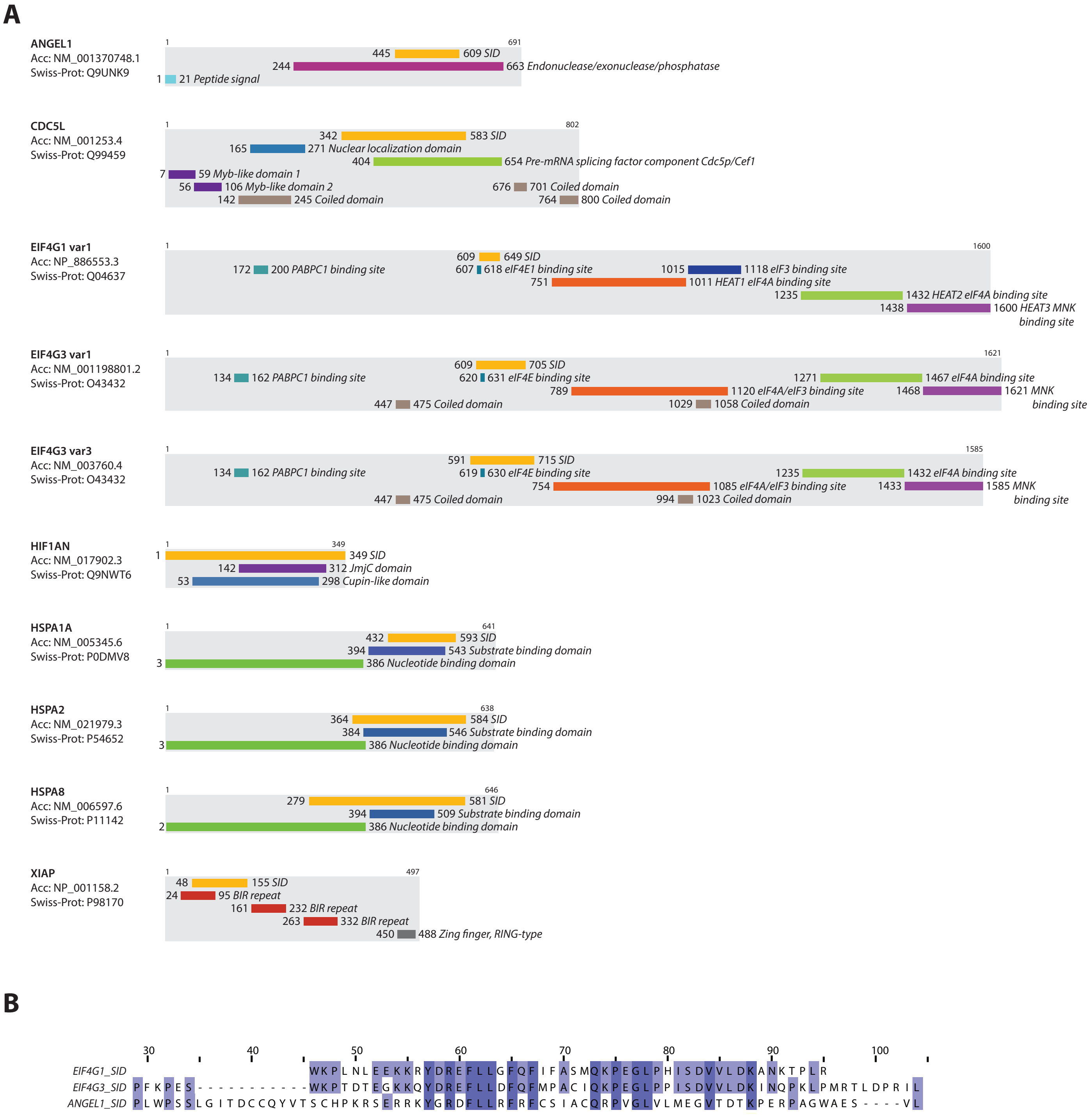

Figure S2. Representation of the Y2H results protein sequences and analysis of the localisation of the selected interaction domain (SID). A. The eIF4E3 partners are represented according to their peptide chain length. The selected interaction domains as determined by Y2H, and the known functional domains of each protein are indicated. The SID is indicated in yellow. **B.** Sequence alignement of the SID identified on Angel1, eIF4G1 and eIF4G3 for eIF4E3 as represented with Jalview.

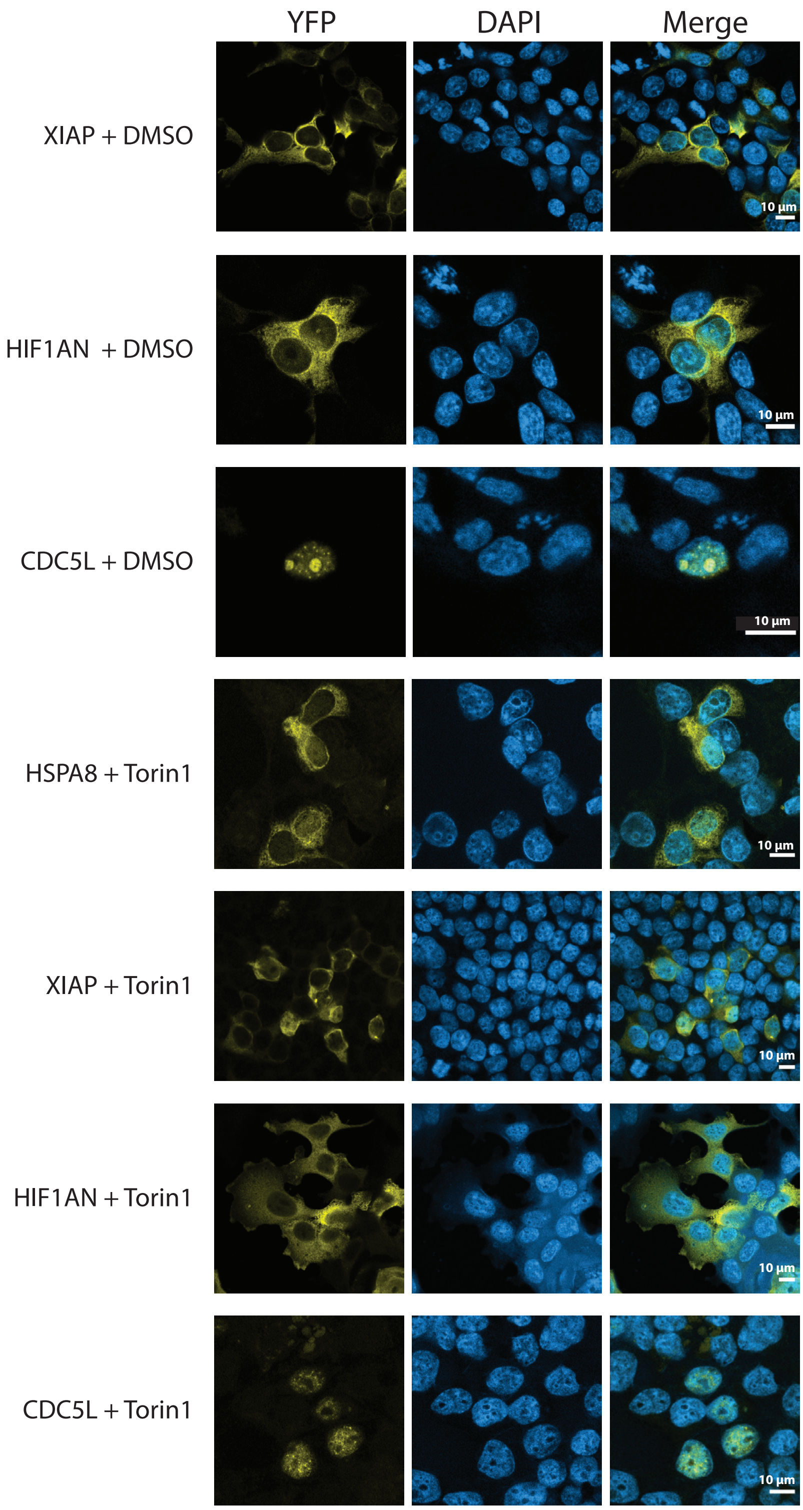

Figure S3. The eIF4E3 partners discovered by Y2H. Bimolecular complementation assay using Venus YFP performed in transfected HEK293T cells treated for 2 hours with DMSO or with 250 nM Torin1 and monitored by confocal microscopy. Venus fragment 1 and Venus fragment 2 were respectively fused to eIF4E3 and to one of the test partners. These are indicated at the side of the left hand panel. The YFP, DAPI and merged images are shown.

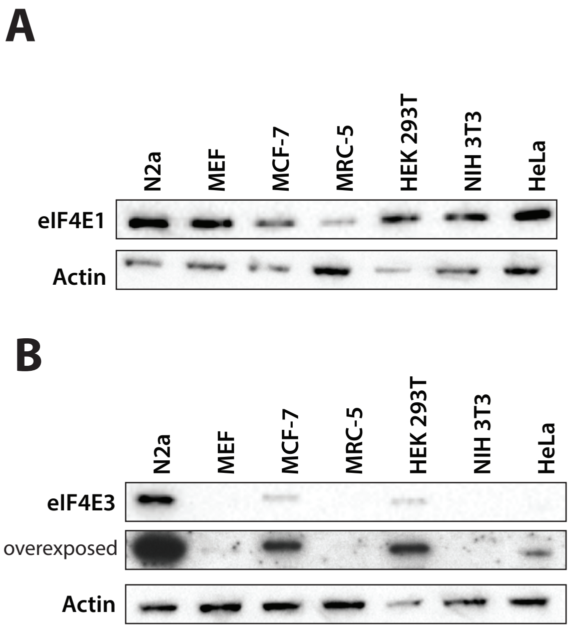

Figure S4. Expression level of eIF4E1 and eIF4E3 in cell lines. Western blots showing the level of expression of eIF4E1 (A) and eIF4E3 (B) in several mammalian cell lines.

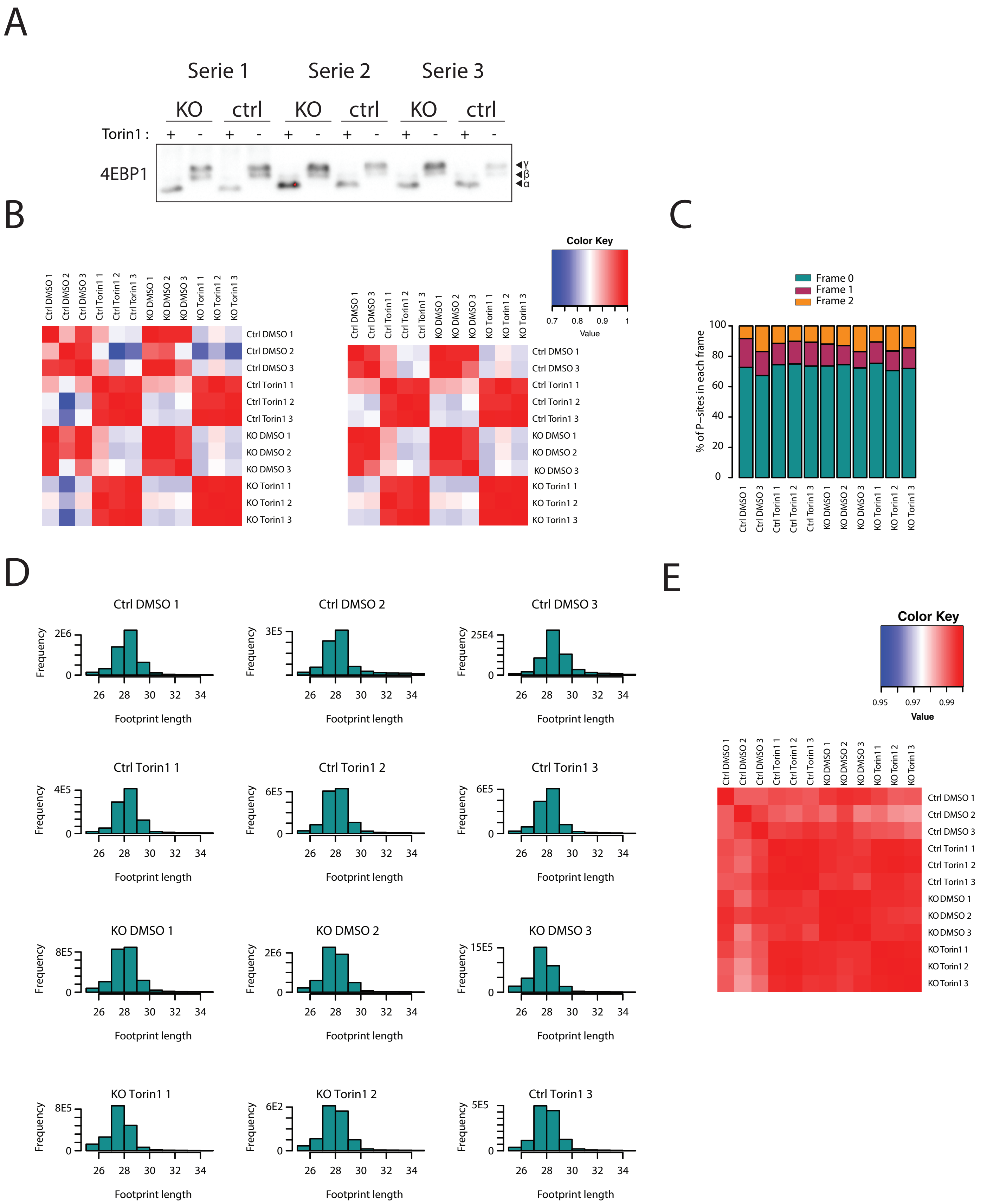

Figure S5. Quality control analysis of the RNA-seq and Ribo-seq data. A. Western blot showing the phosphorylation levels of 4EBP1 after treatment with DMSO or 250 nM Torin1 in the three replicates used for ribosome profiling. **B.** Heatmap of pairwise Pearson correlations of log2 RPKMs of ribosome footprinting data. These compare all individual samples, including (**left**) and excluding (**right**) Ctrl DMSO 2. This sample was removed in all downstream analysis. **C.** Barplot of the percentage of predicted P-sites found in each frame, summed over all CDS. **D.** Histograms of ribosome footprint lengths for each sample. **E.** Heatmap of pairwise Pearson correlations of log2 RPKMs of RNASeq data for all samples.

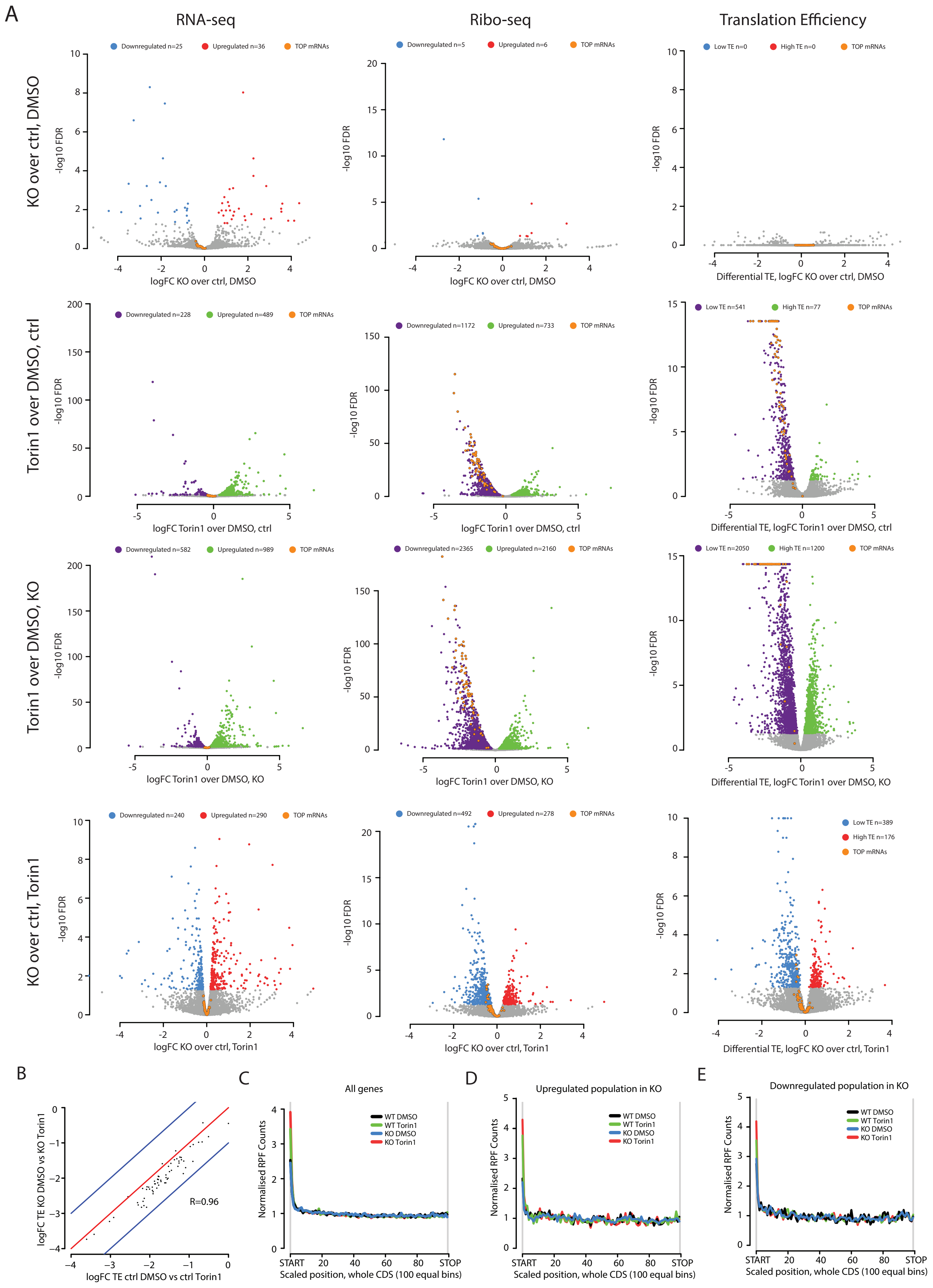

Figure S6. Representation of the RNA-seq, Ribo-seq and translation efficiency data and the eIF4E3 KO only impacts translation at initiation in Torin1 treatment. A. Volcano plots for all relevant pairwise comparisons of condi-

tions (by row). The first, second and third columns show the changes for each comparison in RNA-seq, Ribo-seq and Translational Efficiency, respectively. Transcripts showing significant changes are highlighted, as are TOP mRNAs. **B.** Scatterplot of differential translational efficiency comparing DMSO and Torin1 one in ctrl vs KO. **C-E.** Scaled metagene plots for KO and WT (treated and untreated) for Ribo-seq p-site depth across the CDS for all genes (**C**) and genes up-regulated (**D**) and downregulated (**E**) for RPFs in KO vs ctrl. Every CDS in the gene group was split into 100 equal bins and the number of p-sites falling in each was aggregated. These were normalised by counts for each CDS and total counts genome-wide.

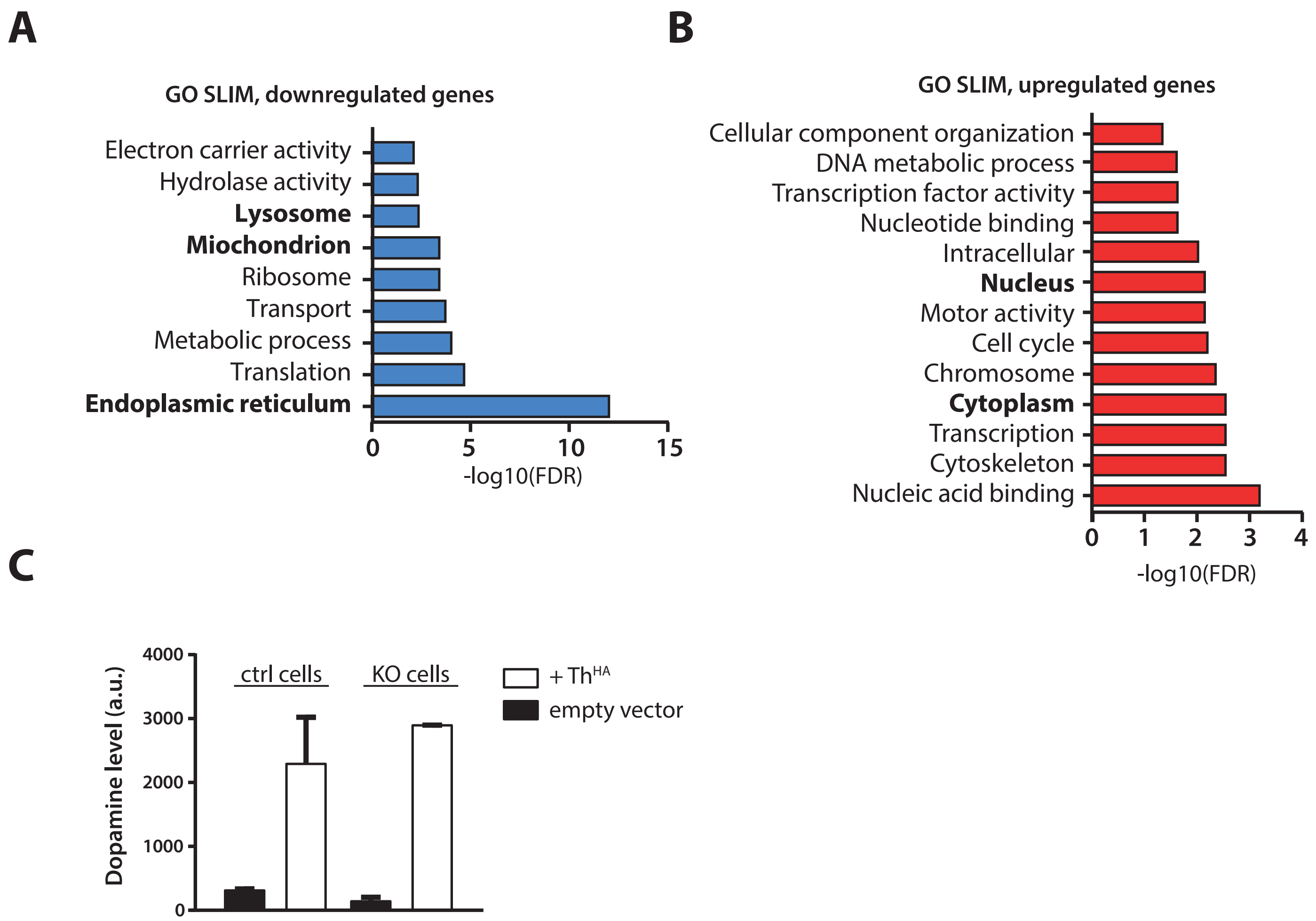

Figure S7. Down and upregulated genes have distinct cellular localisation and the transduction of Th^{HA} restaures the production of dopamine in N2a cells. A-B. Barplot of FDR values for hypergeometric tests showing enrichment of various GO SLIM terms among genes downregulated (**A**) and upregulated (**B**) in KO vs ctrl, in Torin1. **C.** Histogram of the intracellular dopamine level in ctrl and KO N2a cells transduced with empty vector or with Th^{HA}.
